# Gender-specific factors associated with hypertension among women of childbearing age: Findings from a nationwide survey in India

**DOI:** 10.3389/fcvm.2022.999567

**Published:** 2022-12-14

**Authors:** Pragti Chhabra, Shyambhavee Behera, Rahul Sharma, Rajeev Kumar Malhotra, Kedar Mehta, Kritika Upadhyay, Sonu Goel

**Affiliations:** ^1^Department of Community Medicine, University College of Medical Sciences, New Delhi, India; ^2^Delhi Cancer Registry, Institute Rotary Cancer Hospital, All India Institute of Medical Sciences, New Delhi, India; ^3^Department of Community Medicine, GMERS Medical College, Vadodara, India; ^4^Department of Community Medicine and School of Public Health, Post Graduate Institute of Medical Education and Research (PGIMER), Chandigarh, India

**Keywords:** hypertension, non-communicable diseases, women, risk factors, health survey

## Abstract

**Background:**

The association of conventional (modifiable and non-modifiable) risk factors for hypertension has already been established in the literature. However, there are other putative risk factors specific to women (early menarche, age at first childbirth, women empowerment, number of children born, hysterectomy, etc.) in the development of hypertension. This study is the first study to highlight the potential association of gender-specific factors along with other conventional risk factors and hypertension, using a nationwide sample.

**Methods:**

The study is a secondary analysis of the data collected from the National Family Health Survey-4 (NFHS-4), a nationally representative sample of 699,686 women of reproductive age in India. The interview schedule included data on general background characteristics, marriage, reproductive history, hysterectomy, knowledge, and utilization of family planning services, maternal and child care, women empowerment, non-communicable diseases, and domestic violence. The blood pressure was measured by direct observation by the study investigators using a digital blood pressure monitor. To account for disproportionate sampling and non-response, a weighted statistical analysis was performed. Logistic regression analysis was done to study the strength of the association between the risk factors and hypertension (computation of unadjusted and adjusted odds ratio).

**Results:**

The prevalence of hypertension was 11.8% among women. Among the conventional factors, older age, higher body mass index (BMI), tobacco use, and alcohol use had higher odds for hypertension, while higher education, higher socio-economic position, and living in urban areas had lower odds. Among the gender-specific factors, younger age at first childbirth, early menarche, oral contraceptive pill use [adjusted OR: 1.23; (1.18–1.28)], and hysterectomy [adjusted OR: 1.10; (1.05–1.69)] were found to be risk factors for hypertension. Domestic violence was significantly associated with hypertension [unadjusted OR: 1.11; (1.02–1.20)]. Empowered women had lower odds of hypertension [adjusted OR: 0.93; (0.95–1.03)].

**Conclusion:**

Significant association of these gender-specific factors among women necessitates the need for taking into account these factors while screening for hypertension among women and thus, designing a tailored model better suited to them for risk assessment.

## Introduction

India has experienced a rapid transition in context to the disease burden from communicable to non-communicable diseases (NCDs) over the past couple of decades (1). High blood pressure was ranked third among the top 10 risk factors accounting for most of the deaths and disabilities in India, in the year 2019 (2). The great India blood pressure survey reported a high prevalence of hypertension among women (23.7%) (3). The highest number of death and disability reported in women is due to non-communicable diseases, including hypertension (1, 4). Hypertension is also an established risk factor for numerous cardiovascular diseases, such as heart failure, atrial fibrillation, and stroke (5).

Established risk factors for hypertension include modifiable (unhealthy diet, physical inactivity, tobacco and alcohol consumption, and overweight, and obesity) and non-modifiable (age, co-existing morbidities, such as diabetes and kidney disease) risk factors (6). Despite both men and women sharing numerous common risk factors for hypertension, the magnitude, distribution, and diversity of these risk factors vary among men and women (4, 7). Accessibility barriers due to socio-cultural and socio-economic limitations among women also contribute toward medical neglect among them (4). Social customs related to physical activity are probable causes of obesity in women making them more vulnerable to hypertension (8). Also, the programs targeted toward the health of women of reproductive age till now have focused mainly on improving their sexual and reproductive health.

Apart from these conventional risk factors for hypertension, including overweight and obesity, physical inactivity, tobacco consumption, alcohol intake, and cholesterol, studies have reported other putative factors that are specific to women in the development of hypertension, such as age at menarche, history of hysterectomy, age at first childbirth, and domestic violence (9–13). Hypertension is more likely to be present among women following hysterectomy (10). Severe emotional violence was found to be significantly linked with hypertension among women (12). Studies have also reported a significant association between age at menarche and the risk of hypertension (9, 14). Thus, these gender-specific factors including early menarche, age at first childbirth, gestational diabetes, women empowerment, number of children born, hysterectomy, and domestic violence also need to be put forward in conjunction with well-established modifiable risk factors of hypertension and other NCDs.

Owing to the evolving epidemiological transition that is taking place in the country, the National NCD Monitoring Framework has set the target to reduce the prevalence of raised blood pressure to 25% by 2025 (15). Addressing and acting upon these otherwise concealed factors will provide a finer insight into the spectrum of hypertension among women and will further assist in the early identification and management of the disease among them. The importance of a step-wise tool for screening cardiovascular disease in women, including women-specific risk factors and highlighting the need for early screening of risk factors for cardiovascular diseases among them, has also been discussed in the literature (16).

The association of these conventional and gender-specific risk factors with hypertension has previously been quoted in the literature, with different risk factors studied and reported separately in different studies. However, there is a dearth in the literature addressing all of these factors together. In our study, we have tried to address the same, altogether using the data from The National Family Health Survey-4 (NFHS-4) conducted to provide data on health, nutrition, family welfare, and emerging health-related issues. Using data from a nationwide survey, the study aims to explore the association between hypertension and these gender-specific risk factors in women of childbearing age. The study of these gender-specific factors adjusting for the conventional risk factors for hypertension has not been addressed in the previous literature.

## Materials and methods

### Study design and participants

The study was conducted using secondary data from NFHS-4, a nationwide cross-sectional survey that was conducted in the years 2015–2016 in India. The sample size was calculated to compute various health-related indicators which were representative of the population parameter at district, state/union territory, and national levels. Separate evaluations were made for urban and rural areas. A total of 699,686 women completed the survey and were included in the analysis. The data extraction was done with the inclusion of women of childbearing age between 15 and 49 years.

### Sampling and data collection

The National Family Health Survey-4 uses a two-stage cluster random sampling method for data collection. The stratification of the sampling frame was done by separating each district into rural, and urban areas before the selection of primary sampling units (PSUs), i.e., census enumeration block (CEB) in the urban areas and villages in the rural areas. All women between 15 and 49 years of age who stayed over the night before the survey in the selected household were included for data collection for the women’s survey module. The individual response rate for women was 94.5%. Given the non-proportional allocation of samples in different survey domains, sampling weights were computed to ensure the representativeness of the survey results at all levels. Special weight for domestic violence was calculated to account for only one woman per household to be selected randomly for the domestic violence module. Further details of the survey methods including sampling, data collection, and sample size estimation are available in the NFHS-4 country factsheet (8).

The data collection for the women’s module included questions on general background characteristics, marriage, reproductive history, hysterectomy, menstrual hygiene, knowledge, and utilization of family planning services, maternal and child care, fertility preferences, husband’s background, women empowerment, non-communicable diseases, HIV/AIDS, and domestic violence. The blood pressure was measured by direct observation by the study investigators using a digital blood pressure monitor (Omron blood pressure monitor) (8). Three readings of blood pressure were recorded for every participant. The module for domestic violence was applied only to a sub-sample at the state level. A list of local organizations was provided to female victims of domestic violence in case of any help.

### Statistical analysis

The analysis for the present study was done for women of childbearing age between the age of 15 and 49 years. To account for disproportionate sampling and non-response, a weighted statistical analysis was performed. Sample weights specific to women and for the computation of indicators of domestic violence (provided along with the dataset) were used in the analysis. Extreme values or outliers were excluded from the study before the analysis. Stata version 12 software was used for analysis. Cleaned descriptive data were reported, and the categorical data were represented in proportions and continuous data through mean (with standard deviation). In addition to conducting bivariate analyses, a binary logistic regression model was employed to calculate the adjusted odds ratio (OR) with the corresponding 95% confidence interval (CI). Complex sample analysis was done to compute both unadjusted and adjusted ORs to account for the stratified cluster-sampling design of the study. Collinearity between the independent variables was checked at the time of regression analysis. Adjusted ORs were not computed for marital status, and age at first childbirth, given their multicollinearity with other independent variables. Also, the variables, early menarche and domestic violence were excluded from the regression analysis due to the loss of a significant dataset on their inclusion in the regression model. A separate category “unknown” was made for the variable of women empowerment for the women who were either not eligible or had not responded to the questions regarding women empowerment.

Women with hypertension between the age group of 15 and 49 years were taken as the dependent variable. Independent variables were carefully chosen based on their putative association as reported in the literature. Independent variables included the age of the participant, educational status, wealth index, type of place of residence, body mass index, age at first childbirth, the total number of children born, women empowerment, domestic violence, use of oral, and injectable contraceptive.

### Operational definitions used

#### Hypertension

Hypertension was defined as “systolic blood pressure ≥140 mm of Hg or diastolic blood pressure ≥90 mm of Hg or history of current intake of antihypertensive medications.” Hypertension was computed using three variables, i.e., the average of the three systolic and diastolic blood pressure readings, and questions asked regarding the history of intake of any antihypertensive medications. In case of missing readings for any of the systolic or diastolic blood pressure, the average of the available two or one individual reading was taken to compute systolic and diastolic blood pressure.

#### Women empowerment

Empowered woman is one who is currently married who “makes all three specific decisions including own healthcare, large household purchase and visits to family or relatives, either alone or jointly with the husband,” and “disagree with all the specific reasons justifying wife-beating including burning food, arguing with him, going out without telling him, neglecting the children, and refusing to have sexual intercourse with him.”

#### Domestic violence

Domestic violence was defined as “any physical violence experienced since the age of 15 years or any sexual violence ever experienced.”

#### Use of oral/Injectable contraceptives

The use of oral/injectable contraceptives was defined as “ever use of the contraceptive including current and past use.”

## Results

The study involved 699,686 women between 15 and 49 years of age. The overall response rate for the questions about raised blood pressure questionnaire was almost 98%.

### Prevalence of hypertension and other independent study variables

The weighted prevalence of hypertension and distribution of socio-demographic characteristics and other gender-specific factors is shown in Table 1. Hypertension was reported among 11.8% of the study participants. However, 9.7% of the study participants were not previously diagnosed as having hypertension at the time of the interview. The average age of the study participants was found to be 29.9 ± 9.7 years. The average age at menarche was reported to be 13.4 ± 1.1 years, and the average age at first childbirth was 25.4 ± 4.8 years. The response rate for the question asked for “age at menarche” was only 34.7%. However, early menarche, defined as menarche occurring before the age of 12 years, was reported by only 3% of study participants. Domestic violence, defined as any kind of sexual or physical violence, was reported by almost 30% of women.

**TABLE 1:** Characteristics of women aged 15–49 years (weighted).

Sample characteristics		Frequency	Percentage (%)
Hypertension	Present	80,397	11.8
	Absent	602,232	88.2
Age	15–19 years	121,552	17.4
	20–24 years	122,966	17.6
	25–29 years	115,043	16.4
	30–34 years	96,769	13.8
	35–39 years	90,890	13.0
	40–44 years	77,969	11.1
	45–49 years	74,497	10.6
Marital status	Never in union	159,035	22.7
	Currently married	511,373	73.1
	Formerly married	29,279	4.2
Education	No education	192,135	27.5
	Primary	87,233	12.5
	Secondary	331,037	47.3
	Higher	89,281	12.8
Body mass index	Underweight	153,331	22.5
	Normal	390,201	57.1
	Overweight	105,038	15.4
	Obese	34,269	5.0
Wealth Index	I	124,054	17.7
	II	136,900	19.6
	III	143,814	20.6
	IV	147,978	21.1
	V	146,939	21.0
Type of place of residence	Urban	242,225	34.6
	Rural	457,461	65.4
Tobacco consumption	Yes	47,551	6.8
	No	652,135	93.2
Alcohol consumption	Yes	8,638	1.2
	No	691,048	98.8
Age at first child birth	15–19 years	53,428	11.0
	20–24 years	195,904	40.4
	25–29 years	153,857	31.7
	30–34 years	60,970	12.6
	>35 years	20,545	4.2
Total number of children born	No child	213,078	30.5
	1 Child	99,101	14.2
	2 Children	175,433	25.1
	3 Children	106,624	15.2
	4 or more children	105,451	15.1
Early menarche (<12 years)	Present	7,301	3.0
	Absent	235,753	97.0
Women empowerment	Empowered	31,254	6.1
	Not empowered	480,119	93.9
Domestic violence	Present	23,838	29.9
	Absent	55,891	70.1
Ever used oral contraceptive pills	Yes	54,742	7.8
	No	644,944	92.2
Ever used injectable contraceptive	Yes	2,459	0.4
	No	697,227	99.6
Had undergone hysterectomy	Yes	22,053	3.2
	No	677,633	96.8

### Association of hypertension and socio-demographic/conventional factors

All the conventional risk factors including age, education, body mass index, tobacco, and alcohol consumption were found to be statistically significant on bivariate analysis. Also, currently married [OR: 3.74; (3.60–3.89)]/formerly married [OR: 5.71; (5.40–6.05)] women had higher odds of having hypertension than never-married women, on bivariate analysis (Table 2).

**TABLE 2:** Bivariate analysis of conventional and other gender-specific factors for hypertension among women of age 15–49 years (unadjusted odds ratio).

		Prevalence (%)	OR (95% CI)
**Conventional risk factors for hypertension**			
Age	15–19 years	3.1	1
	20–24 years	4.8	1.59 (1.50–1.68)
	25–29 years	7.4	2.53 (2.39–2.67)
	30–34 years	11.5	4.10 (3.89–4.33)
	35–39 years	16.4	6.19 (5.88–6.51)
	40–44 years	22.2	9.08 (8.55–9.48)
	45–49 years	11.8	12.15 (11.54–12.79)
Marital status	Never in union	4.1	1
	Currently married	13.7	3.74 (3.60–3.89)
	Formerly married	19.5	5.71 (5.40–6.05)
Education	No education	15.3	1
	Primary	14.1	0.90 (0.87–0.93)
	Secondary	10.1	0.61 (0.59–0.62)
	Higher	8.5	0.51 (0.49–0.54)
Body mass index	Underweight	6.1	1
	Normal weight	9.8	1.67 (1.62–1.73)
	Overweight	21.2	4.13 (3.98–4.29)
	Obese	30.9	6.90 (6.58–7.23)
Wealth Index^$^	I	10.2	1
	II	10.7	1.05 (1.02–1.09)
	III	11.2	1.11 (1.07–1.15)
	IV	13.0	1.32 (1.27–1.36)
	V	13.4	1.36 (1.31–1.41)
Type of place of residence	Urban	12.8	1
	Rural	11.2	0.86 (0.83–0.88)
Tobacco consumption	No	11.4	1
	Yes	16.6	1.54 (1.48–1.59)
Alcohol consumption	No	11.7	1
	Yes	19.9	1.88 (1.76–2.01)
**Gender-specific factors for hypertension**			
Age at first child birth^#^	15–19 years	12.5	1
	20–24 years	12.9	1.03 (0.98–1.08)
	25–29 years	15.5	1.28 (1.22–1.34)
	30–34 years	18.0	1.52 (1.44–1.61)
	>35 years	19.3	1.66 (1.56–1.77)
Total number of children born	No child	5.2	1
	1 Child	10.4	2.12 (2.02–2.21)
	2 Children	14.0	2.96 (2.85–3.06)
	3 Children	16.3	3.56 (3.43–3.69)
	4 or more children	17.9	3.96 (3.82–4.10)
Early menarche (<12 years)	Absent	3.9	1
	Present	4.6	1.18 (1.00–1.40)
Women empowerment	Not empowered	13.7	1
	Empowered	13.7	0.99 (0.95–1.05)
	Unknown	6.5	0.43 (0.42–0.44)
Domestic violence	Absent	11.0	1
	Present	12.1	1.11 (1.02–1.20)
Ever used oral contraceptive pills	No	11.5	1
	Yes	15.3	1.39 (1.34–1.45)
Ever used injectable contraceptive	No	11.8	1
	Yes	12.6	1.08 (0.90–1.30)
Had undergone hysterectomy	No	11.4	1
	Yes	23.7	2.41 (2.30–2.53)

OR, odds ratio; CI, confidence interval. ^#^Age-adjusted odds ratio: 15–19 years, 20–24 years, 25–29 years, 30–34 years, 35–39 years, 40–44 years, and 40–49 years were found to be: 1 (referent), 0.84 (0.82–0.87), 0.75 (0.73–0.78), 0.68 (0.65–0.70), 0.60 (0.57–0.63), 0.50 (0.45–0.55), and 0.49 (0.36–0.69), respectively (*p*-value <0.001). ^$^Category I denotes the lowest quintile and V denotes the highest quintile.

Older age was reported to be a significant predictor of hypertension in the current study after adjustment for other independent variables. The higher the level of education, the lesser the odds of having hypertension (Table 3). Although higher odds of hypertension were reported among women who were in the higher Wealth Index quintile on univariate analysis, women who belonged to this bracket had lower odds compared to the lower Wealth Index quintile group, after adjusting for the confounders. Women who consumed any kind of tobacco [adjusted OR: 1.09; (1.05–1.13)] or alcohol [adjusted OR: 1.52; (1.41–1.63)] had higher odds of having hypertension (Table 3).

**TABLE 3:** Multivariate regression analysis of conventional and other gender-specific factors for hypertension among women of age 15–49 years.

		Adjusted OR (95% CI)
**Conventional risk factors for hypertension**		
Age	15–19 years	1
	20–24 years	1.55 (1.46–1.66)
	25–29 years	2.33 (2.17–2.50)
	30–34 years	3.53 (3.29–3.79)
	35–39 years	5.14 (4.80–5.51)
	40–44 years	7.31 (6.80–7.84)
	45–49 years	9.76 (9.09–10.48)
Education	No education	1
	Primary	1.04 (1.01–1.08)
	Secondary	0.96 (0.92–0.99)
	Higher	0.78 (0.73–0.82)
Body mass index	Underweight	1
	Normal weight	1.37 (1.32–1.41)
	Overweight	2.63 (2.52–2.74)
	Obese	4.20 (3.98–4.42)
Wealth Index^$^	I	1
	II	0.98 (0.94–1.01)
	III	0.94 (0.90–0.98)
	IV	1.00 (0.96–1.04)
	V	0.94 (0.89–0.98)
Type of place of residence	Urban	1
	Rural	1.06 (1.02–1.09)
Tobacco consumption	No	1
	Yes	1.09 (1.05–1.13)
Alcohol consumption	No	1
	Yes	1.52 (1.41–1.63)
**Gender-specific factors for hypertension**		
Total number of children born	No child	1
	1 Child	0.89 (0.85–0.94)
	2 Children	0.82 (0.78–0.87)
	3 Children	0.83 (0.79–0.88)
	4 or more children	0.79 (0.75–0.84)
Women empowerment	Not empowered	1
	Empowered	0.93 (0.89–0.98)
	Unknown	0.99 (0.95–1.03)
Ever used oral contraceptive pills	No	1
	Yes	1.23 (1.18–1.28)
Ever used injectable contraceptive	No	1
	Yes	0.89 (0.74–1.06)
Had undergone hysterectomy	No	1
	Yes	1.10 (1.05–1.69)

OR, odds ratio; CI, confidence interval. ^$^Category I denotes the lowest quintile and V denotes the highest quintile.

### Hypertension and gender-specific factors

On bivariate analysis, younger age at first childbirth appeared to be a protective factor for hypertension (Table 2). However, as we expected “participant’s age” to be an evident confounder for the variable age at first birth, we performed an age-adjusted analysis for this variable. In doing so, younger age at first childbirth was observed to be a risk factor for hypertension (Table 2, footnote). The association of early menarche was found to be significant with hypertension [unadjusted OR: 1.18; (1.00–1.40)]. Higher odds of hypertension were reported among women who had experienced domestic violence [unadjusted OR: 1.11; (1.02–1.20)].

Empowered women had lower odds of hypertension as compared to women who were not empowered [adjusted OR: 0.93; (0.89–0.98)] (Table 3). Women with a history of oral contraceptive pill use had significantly higher odds of hypertension [adjusted OR: 1.23; (1.18–1.28)]. Women who had undergone a hysterectomy had 1.10 times higher odds of having hypertension (95% CI: 1.05–1.69) (Table 3).

## Discussion

The established modifiable and non-modifiable risk factors of hypertension are often discussed in the literature. The current study was done to emphasize these putative gender-specific factors and their association with hypertension among women of age 15–45 years from a nationwide survey.

Hypertension was found to be present in 11.8% of the women. However, another nationwide study reported a much higher prevalence of 23.7% among women (3). The large gap in the prevalence could be due to variation in the age distribution of the study participants, with a higher representation of older age women in the latter (3). There is a paucity of literature, addressing the prevalence of hypertension among women of childbearing age. Although a lower rate of hypertension was observed among these women, even this rate poses challenges to overall women’s health in terms of increased risk for future cardiovascular diseases as well as a higher risk of hypertensive disorders of pregnancy, further leading to compromise on maternal and fetal outcomes. Also, adverse cardiovascular disease outcome has been observed among women following delivery who had pre-pregnancy hypertension or any hypertensive disorder of pregnancy (17). Older age is a recognized non-modifiable risk factor for hypertension, which was also observed in the current study (18).

A higher level of education was found to be a protective factor for hypertension in this study. Similar findings were stated by a study done among Australian women (19). However, a study done by Gupta et al. (20) in South Asia reported an opposite trend, with hypertension being more in women with higher education. The findings in the present study could be attributed to the moving trends of hypertension in line with the epidemiological transition occurring in the country, leading to trends similar to the developed nations (1).

Belonging to the higher Wealth Index quintile had lower odds of hypertension compared to the lower. Similar findings were reported by Leng et al. (21) in a meta-analysis. Higher odds of hypertension was reported among women living in a rural area, in the current study, after adjusting for other factors. A study done in Southeast Asian countries, including India, Bangladesh, and Pakistan, reported conflicting findings with higher odds of hypertension among urban compared to residents living in rural areas (20). However, reverse trends were also observed from Trivandrum, Kerala, and Dhaka, Bangladesh in the same study (20). Studies have shown a declining urban–rural gap in hypertension over the few past decades with the reversal of the trends, which could be attributed to rapid urbanization in rural areas (22). A significant association of hypertension was reported with behavioral factors including overweight and obesity, tobacco, and alcohol consumption. Similar findings have also been reported in other studies done in India and other countries (23, 24).

Younger age at first childbirth was found to be a risk factor in the occurrence of hypertension among women in the current study. Similar findings were also reported in a study done among women between 18 and 45 years of age and another among post-menopausal women (11, 25, 26). Possible reasons for the above findings could be attributed to earlier exposure to insulin resistance, raised lipids, and other physiological changes that carry atherogenic potential (27). Also, other socio-cultural determinants, such as a decline in physical activity and dietary alterations leading to the attainment of higher BMI, could be possible reasons behind such findings.

This study reported a positive association of hypertension with early menarche. Similar findings were reported by Bubach et al. (14) in a meta-analysis. This may partly be explained by the association of early menarche with obesity. However, on the contrary, a study among Chinese women reported late menarche to be associated with hypertension (9). A possible explanation for the above findings was attributed to exposure to chronic malnutrition among the study participants, which could have led to delayed menarche, and thus its association with hypertension (9). The UK million women research has reported a U-shaped relation between menarche and cardiovascular risk and stroke though not specific for hypertension (28).

In a bivariate analysis, higher odds of hypertension were found among women who reported any kind of physical or sexual violence. Similar findings were reported by Stene et al. (29) in a population-based cohort study. Possible reasons for the findings were reported to be a higher probability of indulgence in substance abuse in presence of such stressors. However, The Nurses’ Health Study II, a prospective cohort study, reported a significant association of hypertension only with severe emotional violence (12). Although our study reported a positive association, computation of odds after adjustment for possible confounders was not done in the study due to a smaller sub-sample of women who enquired about domestic violence.

The current study reported decreasing odds of hypertension among women with increasing parity. In contrast to these findings, other studies have reported either a higher or no risk of developing hypertension with increasing parity (30, 31). The discrepancy in the above findings could possibly be due to the cross-sectional nature of the study design, due to which the long-term effect of parity might have been obscured. A prospective study among peri- and post-menopausal women also reported a positive association between parity and early hypertension among women during the menopausal transition phase (30). Also, in the study among African–American women, although higher parity was found to be associated with higher systolic blood pressure, lower levels of diastolic blood pressure were reported among women with high parity. However, the study did not report the overall effect of parity on hypertension (31).

Women empowerment was reported to be a protective factor for hypertension in the current study. Although there is a paucity of data regarding the association between gender empowerment and hypertension in the literature, plausible reasons for the above findings could be attributed to the accessibility and availability of preventive health promotional services and better health literacy toward disease prevention.

In the current study, there was a significant association between hypertension and the use of oral contraceptives. Similar findings have also been reported by The Lancet women and cardiovascular disease Commission. Although hormonal contraceptives are considered safe for preventing pregnancy, the presence of other risk factors, such as prolonged smoking, pre-existing cardiac diseases, and thrombo-embolic disorders toward cardiovascular diseases and hypertension augments the risk of hypertension among these women (13).

Higher odds of having hypertension were reported in the women who had undergone a hysterectomy in the study. A population-based retrospective cohort study by Ding et al. (10), also reported a greater risk of hypertension among women following a hysterectomy before the age of 50 years. The reason for such an association could be due to a higher risk of getting obese following a hysterectomy, possibly due to lower physical activity or dietary modifications following a hysterectomy (32).

This study is the first study to highlight the potential association between other gender-specific factors and hypertension, using a nationwide sample. The introduction of these factors along with the established risk factors has added to the novelty of addressing risk factors for hypertension among women. Also, robust statistical methods for analysis were employed to address the clustered sampling technique used in the survey.

## Limitations

As the study involved secondary data analysis from a national survey, due to the unavailability of data, other possible factors, such as the history of hypertensive disorders of pregnancy, dose–response relation of oral contraceptive use, history of adverse pregnancy outcomes (abortions and stillbirths), and age at menopause could not be assessed.

## Conclusion and recommendations

Established conventional risk factors have been already incorporated into the screening tools for cardiovascular diseases and hypertension. The current study highlights the potential association between other gender-specific factors and hypertension. Significant association of these factors among women necessitates the need for taking into account these factors while screening for hypertension and other cardiovascular diseases among women and thus, designing a tailored model better suited to them for risk assessment. Also, the study highlights the need for the evaluation of the temporal association of these factors using prospective study designs.

## Data availability statement

Publicly available datasets were analyzed in this study. This data can be found here: https://dhsprogram.com/data/dataset/India_Standard-DHS_2015.cfm?flag=1.

## Ethics statement

This study was done using data available in the public domain, accessible on request with the DHS program. This study was ethically approved by the Institutional Ethics Committee, Postgraduate Institute of Medical Education and Research (PGIMER), Chandigarh, India (PGI/IEC/2021/001139).

## Author contributions

PC conceptualized the manuscript. PC, RS, and SB did the data compilation and drafted the manuscript. RM, RS, and SB did the statistical analysis. KM, KU, and SG critically reviewed and edited the manuscript. All authors reviewed the results and approved the final version of the manuscript.
